# The Yersinia Phage X1 Administered Orally Efficiently Protects a Murine Chronic Enteritis Model Against *Yersinia enterocolitica* Infection

**DOI:** 10.3389/fmicb.2020.00351

**Published:** 2020-03-06

**Authors:** Yibing Xue, Shengjie Zhai, Zijing Wang, Yalu Ji, Gang Wang, Tianqi Wang, Xinwu Wang, Hengyu Xi, Ruopeng Cai, Rihong Zhao, Hao Zhang, Lanting Bi, Yuan Guan, Zhimin Guo, Wenyu Han, Jingmin Gu

**Affiliations:** ^1^Key Laboratory of Zoonosis Research, Ministry of Education, College of Veterinary Medicine, Jilin University, Changchun, China; ^2^College of Clinical Medicine, Jilin University, Changchun, China; ^3^Department of Clinical Laboratory, The First Hospital of Jilin University, Changchun, China; ^4^Jiangsu Co-Innovation Center for the Prevention and Control of Important Animal Infectious Disease and Zoonose, Yangzhou University, Yangzhou, China

**Keywords:** *Yersinia enterocolitica*, bacteriophage, chronic enteritis, oral administration, phage therapy

## Abstract

*Yersinia enterocolitica* is generally considered an important food-borne pathogen worldwide, especially in the European Union. A lytic Yersinia phage X1 (Viruses; dsDNA viruses, no RNA stage; Caudovirales; and Myoviridae) was isolated. Phage X1 showed a broad host range and could effectively lyse 27/51 *Y. enterocolitica* strains covering various serotypes that cause yersiniosis in humans and animals (such as serotype O3 and serotype O8). The genome of this phage was sequenced and analyzed. No toxin, antibiotic-resistance or lysogeny related modules were found in the genome of phage X1. Studies of phage stability confirmed that X1 had a high tolerance toward a broad range of temperatures (4–60°C) and pH values (4–11) for 1 h. The ability to resist harsh acidic conditions and enzymatic degradation *in vitro* demonstrated that phage X1 is suitable for oral administration, and in particular, that this phage can pass the stomach barrier and efficiently reach the intestine *in vivo* without losing infectious ability. The potential of this phage against *Y. enterocolitica* infection *in vitro* was studied. In animal experiments, a single oral administration of phage X1 at 6 h post infection was sufficient to eliminate *Y. enterocolitica* in 33.3% of mice (15/45). In addition, the number of *Y. enterocolitica* strains in the mice was also dramatically reduced to approximately 10^3^ CFU/g after 18 h compared with 10^7^ CFU/g in the mice without phage treatment. Treatment with phage X1 showed significant improvement by intestinal histopathologic observations. Moreover, proinflammatory cytokine levels (IL-6, TNF-α, and IL-1β) were significantly reduced (*P* < 0.05). These results indicate that phage X1 is a promising candidate to control infection by *Y. enterocolitica in vivo*.

## Introduction

Yersiniosis is mainly caused by *Yersinia enterocolitica*, which is a facultative anaerobic Gram-negative bacterium that belongs to the Yersiniaceae family (Adeolu et al., 2016; Saraka et al., 2017). Yersiniosis is the third most common zoonotic disease in New Zealand and the most country of European Union (e.g., Germany and Finland) following campylobacteriosis and salmonellosis (Rosner et al., 2010; Strydom et al., 2019). Pigs are asymptomatic carriers and the main reservoir of the *Y. enterocolitica* strain serotype O3, which is the most frequently reported serotype (89%) in the EU (Drummond et al., 2012). *Y. enterocolitica* cause a range of infections including severe diarrhea, enteritis and mesenteric lymphadenitis, reactive arthritis (ReA), nodular erythema (EN) and septicemia (Rosner et al., 2013). It is worth noting that a lot of studies have found that *Y. enterocolitica* has developed resistance to an increasing number of antibiotics, such as the second-generation quinolones, the third-generation cephalosporins, sulfonamides, and streptomycin, which are recognized as first-line treatments for *Y. enterocolitica* infection. Therefore, there is an urgent need for novel therapeutic agents directed against *Y. enterocolitica*.

Many studies have shown the effectiveness of phages in eradicating various food-borne pathogens in humans and animals (Wall et al., 2010; Abedon et al., 2011). Phage is harmless because it is strict bacterial virus that only specifically recognize, infect, and replicate inside host bacteria (Loc-Carrillo and Abedon, 2011). Additionally, phage usually do not destroy intestinal microbiota by specifically targeting host bacteria. Thus, phage therapy possesses great potential as a substitute or supplement for antibiotic treatment (Casey et al., 2018).

From 2000 to 2019, only several *Y. enterocolitica* bacteriophages have been reported (Leon-Velarde et al., 2016; Leskinen et al., 2016). Phage PY100 was administered orally in a murine model of *Y. enterocolitica*, unfortunately, the treatment using this phage with multiple applications and a high dose did not yield satisfying results (Skurnik and Strauch, 2006; Schwudke et al., 2008). Successful phage therapy for *Y. enterocolitica*, especially via oral administration, has rarely been reported.

In this study, the Yersinia phage X1 (Viruses; dsDNA viruses, no RNA stage; Caudovirales; and Myoviridae) was isolated from sewage by using *Y. enterocolitica* strain and the characteristics of this phage was studied. For determining the therapeutic effect of phage X1 on protecting a murine chronic enteritis model against *Y. enterocolitica* infection, the stable of phage X1 within the gastrointestinal tract with oral application and the bactericidal activity of phage X1 was detected *in vivo*.

## Materials and Methods

### Bacterial Strains

The *Y. enterocolitica* strains used in this study are listed in Supplementary Table S1. The reference strains ATCC 23715 (serotype O8) and CICC 21565 (serotype O3) were purchased from American Type Culture Collection (Manassas, VA, United States) and China Center of Industrial Culture Collection (Beijing, China), respectively. The 49 clinical isolates of *Y. enterocolitica* were kindly provided by the Yunnan Institute of Endemic Disease Control. After sequencing the 16S rRNA gene with the universal primers 27F (5′-AGAGTTTGATCCTGGCTCAG-3′) and 1492R (5′-GGTTACCTTGTTACGACTT-3′) (Hao et al., 2016), identification of O3, O5, O8, and O9 serotypes was carried out with slide agglutination test by using diagnosis monoclonal antibodies against *Y. enterocolitica* (Wantai Biotech Co., Ltd.; Zhengzhou, China). Strains were cultured in LB (lysogeny broth, containing 5.0 g yeast extract, 10.0 g peptone, and 10.0 g NaCl per 1000 mL) medium (Bertani, 2004) or on plates with selective diagnostic CIN agar (Cefsulodin-Irgasan-Novobiocin; Oxoid, United Kingdom) at 37°C for 18–24 h (Orquera et al., 2012). For long-term storage, bacterial cultures were stored in glycerol [3:1 (v/v)] at −80°C.

### Phage Isolation and Characteristics

The bacteriophage was isolated, purified and concentrated according to a previously described method (Gu et al., 2011). ATCC 23715 was used as a host to isolate the phage. Sewage samples were collected from a sewage treatment plant of Changchun City, Jilin Province, China. The phage was purified by the double-layer agar plate method for more than three times. To determine the host range of the phage, spot test and double-layer agar plate methods were used as described previously (Leon-Velarde et al., 2016).

The phage was concentrated as previously described (Synnott et al., 2009) and was spotted on a hydrophilic. Formvar-carbon-coated copper mesh for 2 min and negatively stained with 2% phosphotungstic acid for 30 s, after which the liquid was blotted with filter paper. Phage morphologies were observed under a TEM (H-7650; Hitachi, Japan) at 80 kV.

The multiplicity of infection (MOI) is defined as the ratio of the number of phages to the number of host bacteria (Czajkowski et al., 2015). The host strain was cultured in LB to a logarithmic growth phase (OD_600_ nm = 0.6). The phage and host bacteria (10^9^ CFU/mL) were added to LB medium at different MOIs (10^–7^, 10^–6^, 10^–5^, 10^–4^, 10^–3^, 10^–2^, 10^–1^, or 1) and cultured for 6 h at 37°C with shaking at 180 rpm. Phage titration was measured by the double-layer plate method after the incubation.

The one-step growth curve experiment was conducted with some modifications as explained elsewhere (Xi et al., 2019). The phage was mixed with an ATCC 23715 suspension at an optimal MOI of 1. After centrifugation at 4°C for 5 min (10,000 × *g*), the mixture was suspended in 10 mL of fresh LB medium and incubated at 37°C with shaking (180 rpm). The samples at different time were subjected to phage titer determination.

### Genome Sequencing and Bioinformatics Analysis

The phage genome was extracted from the concentrated phage preparation using a viral genome extraction kit (Omega Bio-tek Inc., Norcross, GA, United States). The genome was submitted to BGI Biotechnology Co. Ltd., for genome-wide sequencing via the Illumina HiSeq platform. Potential open reading frames (ORFs) were predicted and analyzed by BLAST (NCBI) and GeneMark (Jeon et al., 2019), and gene function block diagrams were mapped using CLC Main Workbench version 7.7.3 software (CLC Bio-Qiagen, Aarhus, Denmark) (Ji et al., 2019).

### Phage Stability Assay

Phage stability experiments *in vitro* were performed for thermal stability and pH according to the method described previously (Zhang et al., 2013). Phage suspensions were inoculated in SM buffer (pH range 1.0–13.0) for 1 h at 37°C. In addition, the thermal stability was tested by using a phage suspension (3.75 × 10^6^ PFU/mL) that was incubated at 4, 25, 37, 50, 60, 70, and 80°C for 1 h. Phage titer was determined by double-layer plate assay. After 12 h of fasting, BALB/c mice were euthanized by intravenous injection of Fatal Plus (pentobarbital sodium) (100 mg/kg). The intestinal contents from the stomach, duodenum, jejunum, ileum, cecum, and colon of the mice were harvested. The contents were homogenized in 1 mL of phosphate buffered saline (PBS, pH 7.4) and centrifuged at 4°C for 1 min (14,000 × *g*) to obtain the supernatants (Bosak et al., 2018). The stability of the phage was detected with supernatant of PBS aspirate of corresponding parts of intestinal tract, and the phage titer was measured after incubation at 37°C for 10, 30, 60, and 90 min.

### Antibacterial Effect of Phage *in vitro*

Referring to the previous study (Cheng et al., 2017), early exponential cultures of ZTYSG 21 were infected with the phage at five different MOIs (PFU/CFU ratios of 10^–6^, 10^–4^, 10^–2^, and 1), and the mixture was incubated in tubes with shaking at 180 rpm at 37°C. In addition, the uninfected phage group was set as a negative control group. The colony count of the cultures was measured every hour.

### Ethics Statement

Female BALB/C mice aged 6–8 weeks (18–20 g) were provided by the Experimental Animal Center of Jilin University, Changchun, China. Experimental animals were placed under specific pathogen-free conditions and provided sterile materials (cages, bedding, water, and food). All animal experiments strictly abided by the National Guidelines for Experimental Animal Welfare (Ministry of Science and Technology of China, 2006) and conducted according to the experimental practices and standards approved by the Animal Welfare and Research Ethics Committee at Jilin University (permit number: 20181227087). All efforts were made to minimize suffering.

### Phage Therapy in a Murine Model

To test whether phages could eliminate pathogenic bacteria from the intestinal tract of infected asymptomatic animals, a murine chronic enteritis model was established by previously described methods (Damasko et al., 2005). After being starved for 12 h, the mice were orally administered 0.1 mL ZTYSG 21 by using a feeding syringe. Prior to use, exponential-phase bacteria ZTYSG 21 were washed with PBS and resuspended in PBS to 2.05 × 10^9^ CFU/mL. Phage treatment was orally administered at 6 h post infection. BALB/c mice were randomized into three experimental groups: (i) Bacteria-PBS group of 15 infected mice treated with 0.1 mL of PBS at 6 h post infection; (ii) Bacteria-Phage group of 15 infected mice treated with phage (0.1 mL, 1.95 × 10^9^ PFU/mL) in a single dose at 6 h post infection; and (iii) PBS-Control group of 15 healthy mice treated with 0.1 mL of PBS as the controls. The experimental animals were monitored for clinical manifestation for 6 days. The experiments were repeated three times.

Colon and cecum tissues were collected under sterile conditions for microbiological analysis at the indicated times. Briefly, after 6 h of challenge, three mice were randomly selected from each group every 3 h for euthanasia by intravenous injection of Fatal Plus (pentobarbital sodium) (100 mg/kg) until 24 h post challenge. After 24 h, three mice were randomly selected from each group every 24 h for euthanasia, and the bacterial load was measured until 6 days after the challenge. The removed organs were homogenized in 1 mL of PBS buffer, then serially diluted with PBS, and plated on *Y. enterocolitica* selective medium (CIN) for counting the number of colonies forming units (CFU).

To determine the phage titer in the Bacteria-Phage group, the supernatants from each time point were centrifuged at 4°C for 15 min (12,000 × *g*) and filtered. The phage titer was determined by agar double-layer plate experiment (Bosak et al., 2018; Jeon et al., 2019).

To assess histological changes, the intestinal tissue samples harvested from euthanized mice were fixed in 10% zinc formalin and stained with hematoxylin and eosin (H&E). Representative tissue sections from each group were imaged. Tissues in cecum were scored blindly for inflammation and pathology using a 13-point system as described before (Barthel et al., 2003). The scoring included submucosal edema (0–3), polymorphonuclear granulocytes (PMN) infiltration into the lamina propria (0–4), goblet cells (0–3), and epithelial integrity (0–3).

Proinflammatory cytokines (TNF-α, IL-6, and IL-1β) in cecum, colon, and spleen tissues were measured. Briefly, three mice were randomly selected from each group at 12, 24, 48, and 72 h after infection. The removed organs were homogenized in 1 mL of PBS buffer, serially diluted with PBS and detected by an enzyme-linked immunosorbent assay kit (BioLegend, San Diego, CA, United States) (Cai et al., 2013).

### Statistical Analyses

SPSS version 13.0 software (SPSS, Inc., Chicago, IL, United States) was utilized for the statistical analysis. All experimental data were analyzed by one-way analysis of variance (ANOVA). Error bars represent standard deviation of the mean. *P* < 0.05 was considered statistically significant. ^∗^*P* < 0.05; ^∗∗^*P* < 0.01; and ^∗∗∗^*P* < 0.001.

## Results

### Characterization of *Y. enterocolitica* Strains

A total of 49 clinically isolated strains were identified as *Y. enterocolitica* by 16S rRNA sequencing. BLAST analysis showed that the 49 isolates were more than 99% identity to *Y. enterocolitica* strains identified and confirmed in the GenBank database (such as KC776767.1 and LR134492.1). The dominant bacterial serotype was O3 (40%, 18/49), followed by O8 (29%, 13/49), O5 (18%, 8/49), O9 (11%, 5/45), and NT (11%, 5/49) (Supplementary Table S1).

### Isolation and Characterization of Phage X1

**FIGURE 1:**
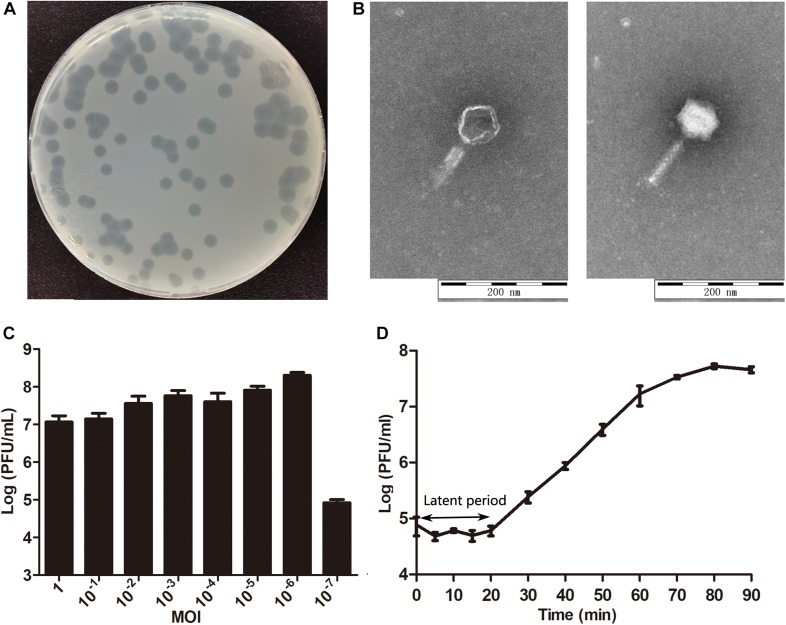
Characteristics of phage X1. **(A)** Plaques induced by phage X1. **(B)** The morphology of phage X1. Transmission electron microscopy (TEM) of phage X1 negatively stained with 2% phosphotungstic acid. The scale bars represent 100 nm. **(C)** Titers of phage X1 under different MOIs in 6 h. When the MOI was 10^–6^, the titer reached the highest value (2.75 × 10^8^ PFU/mL). **(D)** One-step growth curve of phage X1 with a latent period of 20 min. The phage titer increased rapidly within 70 min and stabilized at 80 min. The titers of samples were measured at different time points. The values represent the mean and standard deviation (SD) (*n* = 3).

The phage isolated from the sewage was purified by a double-layer agar plate method and designated Yersinia phage X1 (Viruses; dsDNA viruses, no RNA stage; Caudovirales; and Myoviridae). Plaques of phage X1 were 5–7 mm in diameter surrounded by transparent haloes in the periphery that expanded with time (Figure 1A). As determined by TEM (Figure 1B), phage X1 had an icosahedral head of 70 ± 3 nm and a contracted tail of 90 ± 3 nm in length and belonged to Myoviridae. The host range analysis demonstrated that phage X1 possessed a broad host range and infected 27 (27/51) strains of *Y. enterocolitica* (2 standard strains and 25 clinical isolates), which included pathogens of the following various serotypes: O3 (14/18), O5 (3/8), O8 (6/13), and NT (4/5) (Supplementary Table S1). When the MOI was 10^–6^, the phage titer reached the highest level, approximately 2.75 × 10^8^ PFU/mL (Figure 1C). The one-step growth curve results (MOI = 1) indicated that the latent period of phage X1 infection was 20 min. The burst size was approximately 290 particles/infected cell (Figure 1D).

### General Features of the Genome of Phage X1

**FIGURE 2:**
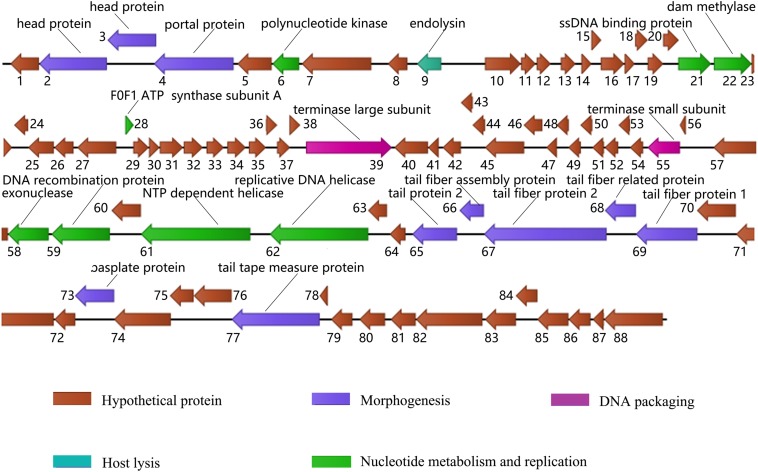
Genome map of phage X1. ORFs are depicted by arrows according to the direction of transcription, and the direction of the arrows represents the direction of gene transcription. Proposed modules are color-coded according to the predicted function of their products. The putative functions and names of the genes are listed above.

Whole genome sequencing of phage X1 is available in GenBank (MN617773). The genome of phage X1 was 48,948 bp in length, with a G + C content of 47.9%. It was found to have 88 putative ORFs, which included 21 proteins of known putative function and 67 hypothetical proteins (Figure 2). ORFs 10–23 and 28–39 were observed to be transcribed on the positive strand and the remaining ORFs were transcribed on the negative stranded. The predicted functional proteins were divided into five modules according to their functions: hypothetical function, structural composition, DNA packaging, host lysis, and nucleic acid metabolism and replication. The host lysis module was the smallest, with only ORF 9 encoding endolysin. No genes related to drug resistance, lysogeny or bacterial virulence were found in the predicted functional genes of phage, at least based on the limited studies.

BLAST analysis showed that the whole genome sequence of phage X1 shows 99.50% sequence identity and 99% genomic coverage with PY100. However, there are differences between X1 and PY100. The size of PY100 genome is 50,291 bp which contains 93 putative ORFs. While, the genome of phage X1 is 48,948 bp and it contains 88 putative ORFs. Though a large part of the ORFs in phage X1 and PY100 show high identity with each other, 10 ORFs in phage X1 shows low query cover and identity with the corresponding putative ORFs that derived from PY100 (as seen from the Supplementary Table S2). It is worth noting that, comparing with PY 100, phage X1 lacks a head protein and an HNH endonuclease which was encoded by ORF58 and ORF91 of PY100, respectively. And four other hypothesis ORFs of PY100 were not found in X1. Additionally, the F0F1 ATP synthase subunit A which encoded by ORF28 of X1 was not found in PY100.

### Stability of Phage X1

**FIGURE 3:**
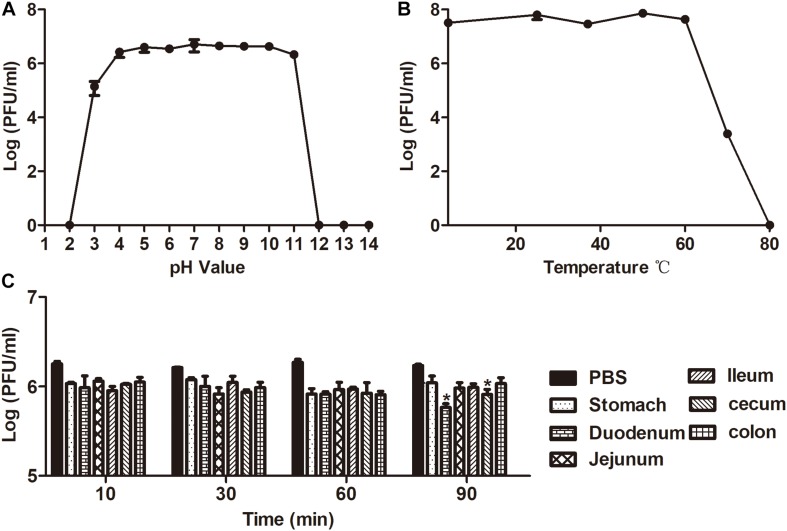
Analysis of the stability of phage X1. **(A)** Stability of phage X1 in different pH values of PBS during 1 h. **(B)** Stability of phage X1 performed at 4, 25, 37, 50, 60, 70, and 80°C during 1 h. **(C)** Stability of phage X1 in gastrointestinal tract content. These values represent the mean and standard deviation (SD) (*n* = 3). *P < 0.05.

The lytic capability of the phage was stable at pH 4–11 for 1h, and almost no loss of activity was observed (Figure 3A). In addition, the sensitivity of phage X1 to various temperatures (4–80°C) is represented in Figure 3B. The activity of phage X1 was stable between 4 and 60°C, although phage activity could be maintained after incubation at 70°C for 1 h. Briefly, we found that phage X1 was highly stable over a wide pH and temperature ranges. Similarly, no significant loss of phage activity was observed when phage X1 was incubated with the intestinal contents at 37°C for 60 min, indicating that these phage particles remained stable under gastrointestinal conditions (Figure 3C).

### Antibacterial Effects of Phage X1 *in vitro*

**FIGURE 4:**
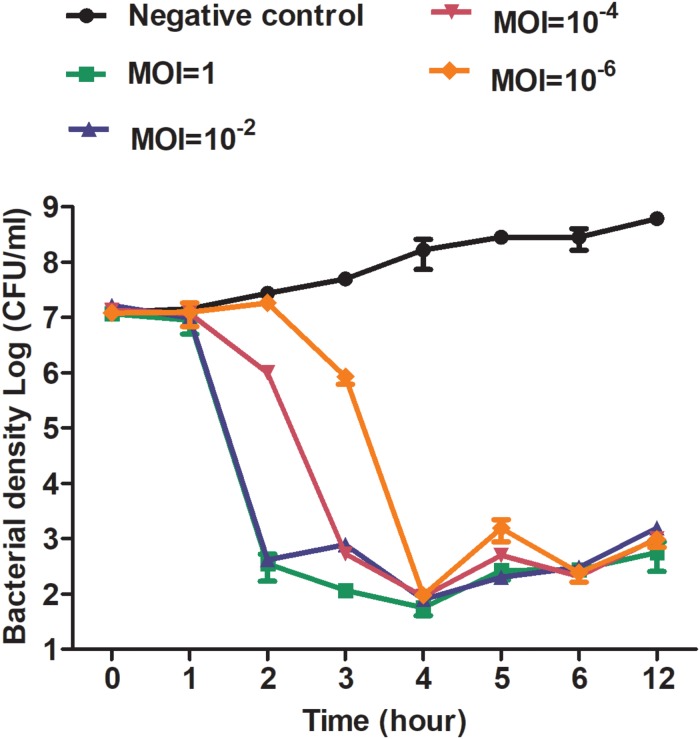
Antibacterial curve of phage X1 *in vitro*. Bacteriolytic activity of phage X1 at different MOIs. ZTYSG21 was infected with phage at the indicated MOI. The colony counts of the negative control group (phage uninfected) and the phage-infected group were determined. The values represent the mean and SD (*n* = 3).

The activity of phage X1 inhibiting ZTYSG 21 growth *in vitro* is shown in Figure 4. As we expected, colony forming unit number increased continuously in the negative control. In contrast, all phage-infected groups dramatically inhibited bacterial growth, although the antibacterial effect was slightly different under different MOIs. With higher MOI values, the bacterium was more sensitive to phage infection. After 4 h, the bacterial counts declined to approximately 10^3^–10^4^ CFU/mL and remained stable compared to 10^8^ PFU/mL in the negative control group.

### Therapeutic Effect of Phage X1 *in vivo*

**FIGURE 5:**
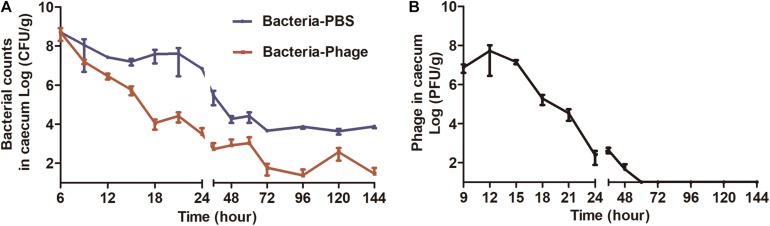
Bacterial and phage loads in the cecum from each group. **(A)** Bacterial load in cecum (CFU/g tissue). Mice infected with ZTYSG21 were sacrificed at the pre-determined times, and 10-fold serial dilutions of cecum homogenates were plated on CIN media to determine bacterial load. **(B)** The titer of the Bacteria-Phage group was observed in the cecum (PFU/g tissue). The experiments were repeated three times. The values represent the means and standard deviations (SDs) (*n* = 3).

The mice challenged with ZTYSG 21 did not die of infection. Therefore, the bacterial loads in the murine intestinal tract were measured. A single oral administration of phage X1 at 6 h post infection was sufficient to eliminate *Y. enterocolitica* in 33.3% of mice (15/45). The Bacterial-PBS group had a median bacterial load in the cecum and colon of 3.84 × 10^7^ CFU/g and 3.79 × 10^7^ CFU/g, respectively, at 18 h (Figure 5A and Supplementary Figure S1A). The treatment with phage X1 significantly decreased the bacterial load to 3.13 × 10^3^ CFU/g and 2.15 × 10^3^ CFU/g, respectively. After 24 h of infection, bacterial counts in the tissues decreased to approximately 10^2^ CFU/g (the untreated mice were approximately 10^4^CFU/g). No colonies were found in the intestine of PBS group mice. Phage X1 had transient increase after oral administration with a slight uptick of the PFU value in the cecum (Figure 5B) and colon (Supplementary Figure S1B). The phage was completely cleared after 48 h of infection. It is worth noting that *Y. enterocolitica* also remained in mice, while phage X1 was cleared by the host immune system. Ten colony-forming units isolated from the germ-carrying mice in the Bacteria-Phage group were randomly selected and propagated. Interestingly, the isolated *Y. enterocolitica* strains were lysed by phage X1 suspension *in vitro*.

**FIGURE 6:**
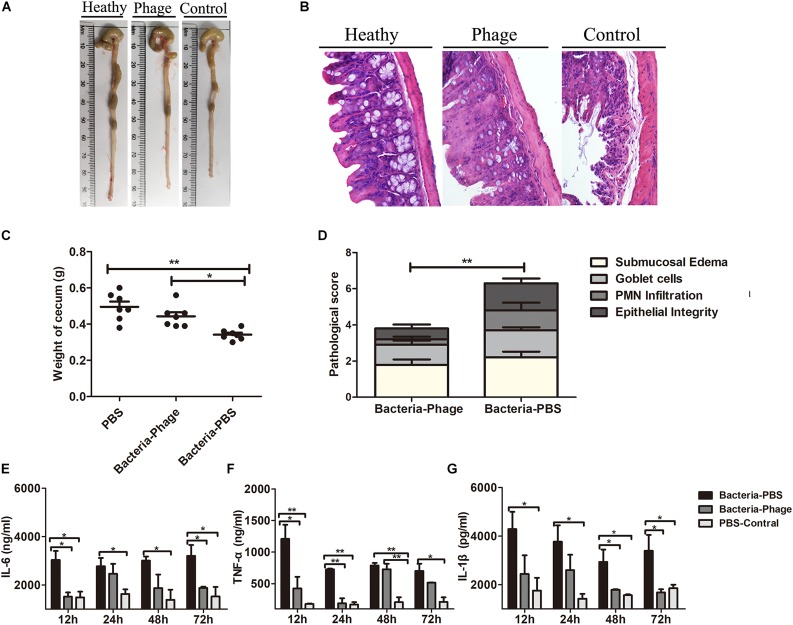
Phage X1 mitigates intestinal inflammation. Representative macroscopic **(A)** and corresponding microscopic images of cecum tissues stained with H&E **(B)**. Images are shown at magnifications of 400. Healthy, phage and control represent the PBS-Control group, Bacteria-Phage group, and Bacteria-PBS group, respectively. **(C)** The weight of the cecum (*n* = 7) and **(D)** histopathology score were measured (*n* = 9). **(E–G)** Production of proinflammatory cytokines in cecum lymphocytes. At 12, 24, 48, and 72 h post infection, the levels of TNF-α, IL-6, and IL-1β in the cecum were determined. The tissues of the healthy mice served as controls. The values represent the means and standard deviations (SDs) (*n* = 3). *P < 0.05; **P < 0.01.

At necropsy, gross and histopathologic lesions in cecum from the different groups were examined. At 48 h post-infection, *Y. enterocolitica* infection induced cecum shortening and increased loss of cecal weight, accompanied by reduced colon size (Figures 6A,C). Microscopically, mice in the Bacteria-PBS group displayed greater cecal histopathology scores than mice in the Bacteria-Phage group (Figure 6D), represented by a loss of goblet cells, mucosal ulcerations, and odema in the submucosa (Figure 6B) and PMN were also observed in the lamina propria of the Bacteria-PBS group mice. In contrast, no significant lesions were observed in the phage-treated mice. The pathological changes were remarkably alleviated in response to X1 treatment.

We also measured the inflammation status in the animals. The levels of the proinflammatory cytokines TNF-α, IL-6, and IL-1β in the cecum (Figures 6E–G), spleen (Supplementary Figure S2A) and colon (Supplementary Figure S2B) of the Bacteria-Phage group were close to the level of healthy mice, whereas they were significantly lower than those of the Bacteria-PBS group (*P* < 0.05). Taken together, these results indicated that phage X1 could mitigate injury in the cecum against *Y. enterocolitica* infection in mice.

## Discussion

Phage treatment for gastrointestinal diseases by oral administration has been studied for a long time. Due to sensitivity to acidic compounds, digestive enzymes, and mucus layer containing IgA antibodies in the gastrointestinal tract, most phages showed limited effect during oral application (Zelasko et al., 2017). The stability of phages under various conditions is an important factor in evaluating their bactericidal potential. Currently, encapsulation of phages by liposomes (Colom et al., 2015) or alginate nanoparticles (Ma et al., 2016) may also be used to enhance bacteriophage stability and bactericidal activity. The pH in the stomach of mice is 3.0 (fed) and 4.0 (starved) (McConnell et al., 2008). In fact, it was found that phage X1 was stable at pH 4–11 and upon incubation with intestinal contents for 1 h, indicating that phage X1 may be suitable for oral administration treatment without packaging technology, making it a cost-effective, convenient, and quick treatment.

The MOI, short latency period and big burst size indicated that phage X1 has high replication efficiency. Phage X1 exhibited a broad host range with at least four pathogenic serotypes (O3, O5, O8, and O9), which especially included O3 serotypes with epidemiological significance (Fredriksson-Ahomaa et al., 2012). Bacteriophage phiYeO3-12 isolated by Pajunen et al. (2001) was only able to infect the O3 serotype of *Y. enterocolitica*. Phage PY54 displays specificity for the O5 serotype of *Y. enterocolitica* (Hertwig et al., 2003). Bacteriophage vB_YenP_AP5 is able to infect serotypes O3, O2, and O1 (Leon-Velarde et al., 2014). Phage PY100 had a broad host range as X1 in the genus *Yersinia* (Schwudke et al., 2008). Though the genome of phage X1 shows high identity with PY100, there are obvious differences between two phages, which may lead to different biological properties and therapeutic effects. ORF67 in phage X1 genome encodes tail fiber protein (TFP), which showed 91% identity with that of PY100. TFP is responsible for the specific initial recognition of host bacteria (He et al., 2018). Therefore, this discrepancy may lead to different lytic activities. There is a *pac* site in terminase small subunit (terS) of phage X1, just like PY100. The risk of transduction is existing due to this site (Djacem et al., 2017).

In a preliminary animal experiment, 10 strains, including the host bacterial strain ATCC 23715, were chosen to infect mice. Only ZTYSG 21 has the longest colonization time in the mouse intestine and does not result in mouse death (Supplementary Table S3). An asymptomatic chronic intestinal infection model in BALB/c mice was established by ZTYSG 21. The murine chronic enteritis model was used to determine the therapeutic effect of phage X1 at a single dose by oral on protecting a murine chronic enteritis model against Y. enterocolitica infection. Phage X1 could lower the number of infected animals and significantly inhibit bacterial growth. Though the phage particles of PY100 were also active for at least 24 h within the gastrointestinal tract (titers between 10^4^ and 10^6^ PFU/g of organ and feces, respectively), PY100, even multiple doses of treatment, did not remove or prevent *Y. enterocolitica* from colonizing the intestines of mice (Skurnik and Strauch, 2006). The newly identified antibacterial agents enterocoliticin (2005) and colicin FY (2018), which remained stable in a gastrointestinal environment, also failed to prevent colonization of *Y. enterocolitica* in the gastrointestinal tract (Damasko et al., 2005; Bosak et al., 2018). Phage X1 also showed higher bactericidal efficiency *in vivo* than *Y. enterocolitica* antimicrobial agents discovered in recent years.

The level of proinflammatory cytokines in the Bacteria-Phage group was significantly decreased compared with the PBS-treated group (*P* < 0.05). Nevertheless, the level of TNF-α in the spleen of the treatment group was still high at 72 h. A number of studies have shown that TNF-α also has an anti-inflammatory effect in limiting inflammation *in vivo* and autoimmune diseases (Mayordomo et al., 2018).

It is conceivable that phage X1 may also be effective in combating bacterial contamination of food products and in veterinary feed. The U.S. Food and Drug Administration (FDA) approved the administration of phages to replace traditional antibiotics in veterinary medicine and as a safe food additive in food products (Bren, 2007). A large number of studies have shown that phages are effective as biocontrol agents against food pathogens and food spoilage organisms (Guenther et al., 2012). Therefore, in our follow-up studies, we will continue to explore the application of phage X1 for the control of *Y. enterocolitica* on food products, as this study demonstrated the high stability and bactericidal efficacy of this phage.

## Data Availability Statement

The datasets generated for this study can be found in the MN617773.

## Ethics Statement

The animal study was reviewed and approved by the Animal Welfare and Research Ethics Committee at Jilin University (permit number: 20181227087).

## Author Contributions

JG, WH, and ZG conceived and designed the study. YX, SZ, ZW, YJ, GW, TW, XW, HX, RC, RZ, HZ, LB, and YG performed the laboratory testing. YX, SZ, and ZW were responsible for the writing and revision of the manuscript. All authors read and approved the final manuscript.

## Conflict of Interest

The authors declare that the research was conducted in the absence of any commercial or financial relationships that could be construed as a potential conflict of interest.
