# Supplementary material for: The Yersinia Phage X1 Administered Orally Efficiently Protects a Murine Chronic Enteritis Model Against Yersinia enterocolitica Infection
Source: Front Microbiol. 2020 Mar 6;11:351. doi: 10.3389/fmicb.2020.00351 (PMC7067902; doi:10.3389/fmicb.2020.00351)

**SUPPLEMENTAL MATERIALS**

**Table S1. Host-range analysis of Yersinia phage X1 against *Y. enterocolitica* strains.**

| **Strain no.^a^** | **Bacterial strain** | **Serotype^b^** | **Spot test^c^** | **Infectivity^c^** |
| --- | --- | --- | --- | --- |
| 1 | HQ119 | O3 | + | + |
| 2 | HQ147 | O9 | - | - |
| 3 | HQ185 | O8 | - | - |
| 4 | HQ239 | O8 | + | + |
| 5 | JC115 | O5 | + | + |
| 6 | JC121 | O3 | + | + |
| 7 | JC174 | O3 | + | + |
| 8 | ZTYS22 | O3 | + | + |
| 9 | ZTYS27 | NT | + | + |
| 10 | ZTYS39 | O8 | - | - |
| 11 | ZTYSG21 | O3 | + | + |
| 12 | ZTYSG9 | O3 | + | + |
| 13 | EY76 | 05 | - | - |
| 14 | LJ14 | O3 | + | + |
| 15 | LJ189 | O5 | + | + |
| 16 | LJ249 | O5 | - | - |
| 17 | LJ257 | O9 | - | - |
| 18 | LJ289 | O3 | - | - |
| 19 | LJ300 | O3 | + | + |
| 20 | LJ330 | O3 | + | + |
| 21 | LJ81 | O8 | + | + |
| 22 | LJGC11 | O9 | - | - |
| 23 | LJGC16 | 09 | - | - |
| 23 | LJGC78 | O9 | - | - |
| 25 | LJYL83 | O5 | - | - |
| 26 | LJYL146 | NT | - | - |
| 27 | LJYL201 | O8 | + | + |
| 28 | LJYL206 | O8 | - | - |
| 29 | LJYL207 | NT | + | + |
| 30 | LJYL251 | O3 | + | + |
| 31 | LJYL252 | NT | - | - |
| 32 | LJYL255 | O3 | - | - |
| 33 | LJYL258 | O8 | + | + |
| 34 | LJYL256 | O3 | + | + |
| 35 | LJYL361 | O3 | + | - |
| 36 | LJYL362 | NT | + | + |
| 37 | ZTYSG8 | NT | + | + |
| 38 | ZTYSG149 | O3 | + | + |
| 39 | ZTYS4 | O3 | + | + |
| 40 | ZTYS8 | O8 | + | + |
| 41 | ZTYS18 | O8 | - | - |
| 42 | ZTYS19 | O3 | - | - |
| 43 | ZTYS21 | O5 | - | - |
| 44 | ZTYS38 | O5 | + | + |
| 45 | ZTYS179 | O8 | - | - |
| 46 | ZTYS197 | O8 | - | - |
| 47 | ZTYS210 | O5 | + | - |
| 48 | ZTYS213 | O3 | - | - |
| 49 | ZTYS214 | O8 | + | - |
| 50 | 23715 | O8 | + | + |
| 51 | 21565 | O3 | + | + |

^A—^ The bacterial hosts were used for the analysis; 1-49, clinical isolates. 50-51, standard strains.

^B—^Serotype identification; NT, Non type, does not belong to *Y. enterocolitica* O3, O5, O8, or O9 serotypes

^C—^ Spot test, Infectivity; +, clear plaque; -, no plaque.

**Table S2. Differential Protein ORFs Between Yersinia phage X1 and PY00**

| Source | ORF NO.^a/b^ | Potential function | Query cover ^c^ | Identity ^c^ |
| --- | --- | --- | --- | --- |
| X1/PY100  X1/PY100  X1/PY100  X1/PY100  X1/PY100  X1/PY100  X1/PY100  X1/PY100  X1/PY100  X1/PY100  X1/PY100  X1/PY100  X1/PY100  X1/PY100  X1/PY100  X1/PY100  X1/PY100 | 8/50  9/49  45/12  46/11  47 /10  50/6  58/92  61/88  67/81  73/74  28/-  -/58  -/91  -/7  -/47  -/51  -/87 | hypothetical protein  Endolysin  hypothetical protein  hypothetical protein  hypothetical protein  hypothetical protein  Exonuclease  NTP dependent helicase  tail fiber protein 2  baseplate protein  F0F1 ATP synthase subunit A  head protein  HNH endonuclease  hypothetical protein  hypothetical protein  hypothetical protein  hypothetical protein | 57%  100%  100%  100%  100%  98%  99%  91%  91%  88%  -  -  -  -  -  -  - | 98%  93%  90%  77%  95%  67%  95%  100%  99%  100%  -  -  -  -  -  -  - |

^a/b—^The preceding ORF: a, X1; b, PY100.

^c—^ The predicted ORFs of X1 query cover and identity comparing with corresponding proteins of PY 100.

**Table S3. Colonization ability of different *Y.enterocolitica* strains.**

| Strains | Bacteria Count in ceacum (CFU/mL) | | | |
| --- | --- | --- | --- | --- |
|  | 24h | 48h | 96h | 144h |
| ZTYSG38  ZTYSG21  ZTYS4  ZTYSG149  LJYL201  JC121  ATCC23715  CICC21565  HQ239  HQ119 | 1.12 × 10^6^  1.10×10^6^  ^_^  1.36× 10^6^  2.10× 10^6^  3.24× 10^3^  1.28× 10^6^  7.90× 10^3^  ^_^  5.62× 10^3^ | 4.51× 10^3^  3.21 ×10^4^  ^_^  4.16× 10^2^  1.40× 10^4^  7.70× 10^2^  9.26× 10^2^  ^_^  ^_^  ^_^ | 5.56× 10^2^  6.59× 10^3^  _  _  5.12× 10^3^  _  _  _  _  _ | _  1.32× 10^4^  _  _  3.71× 10^2^  _  _  _  _  _ |

- No *Y.enterocolitica* bacteria was detected in the cecum

**Fig. S1. Bacterial load and phage titre in the colon.** (A) Bacterial loads in colon (CFU/g tissue). (B) The titres of the Bacteria-phage group were observed in the colon (PFU/g tissue). The experiment was conducted in triplicate, and the results represent the average of the three experiments.


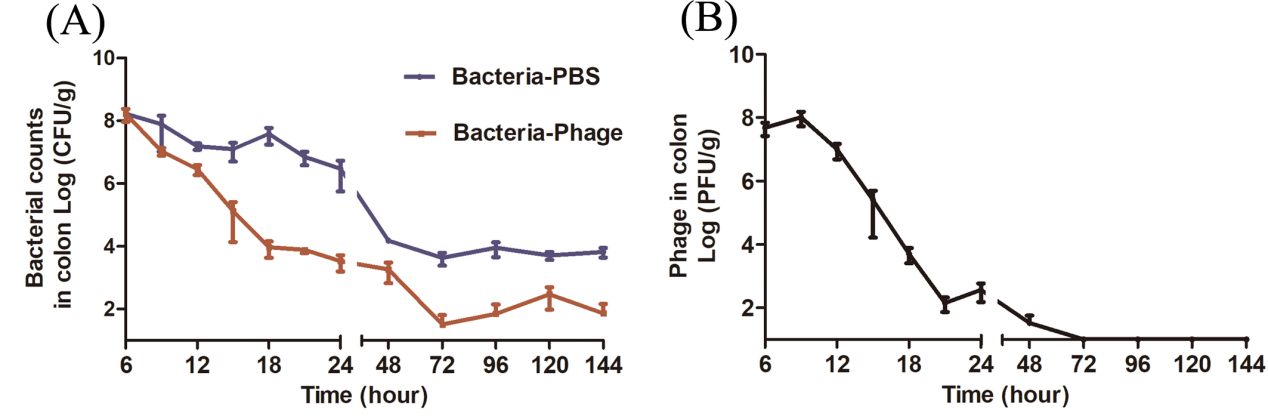


**Fig. S2. Cytokine Analysis.** Generation of proinflammatory cytokines in the spleen (A) and colon (B). At 12, 24, 48 and 72 h post infection, the levels of IL-6, TNF-α and IL-1β in the caecum were determined. The tissues of the healthy mice served as controls. The experiment was conducted in triplicate, and the results represent the average of the three experiments. *, *P* < 0.05; **, *P* < 0.01; ***, *P* < 0.001.


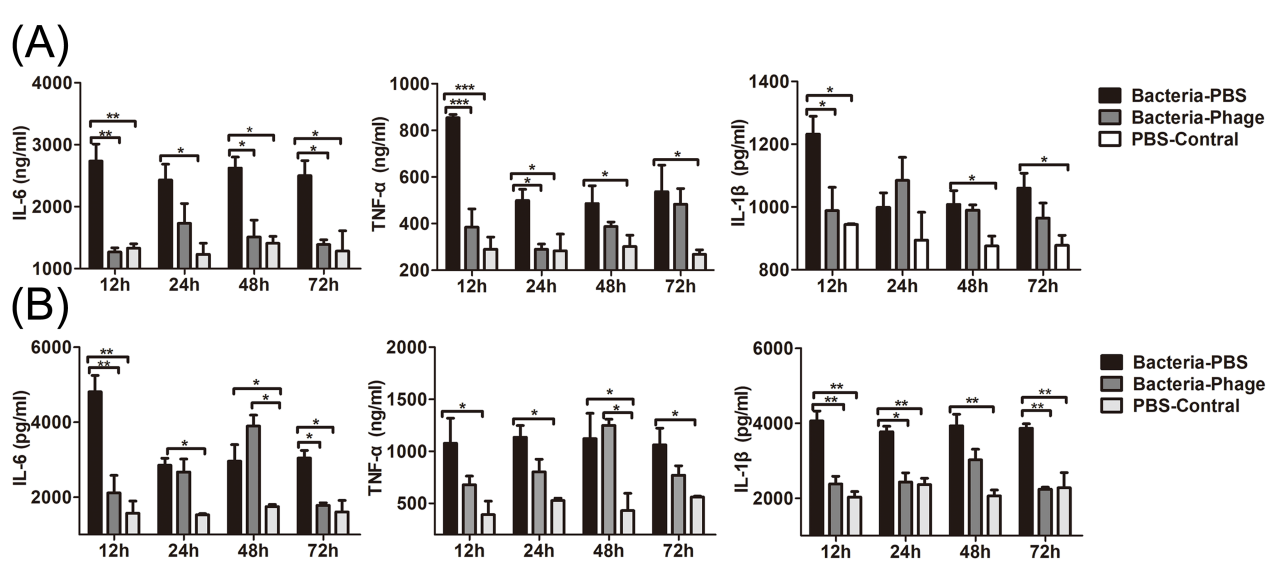

Supplement: Supplementary file 1 [file Data_Sheet_1.docx]
